# Iron-Uptake Systems of Chicken-Associated *Salmonella* Serovars and Their Role in Colonizing the Avian Host

**DOI:** 10.3390/microorganisms8081203

**Published:** 2020-08-07

**Authors:** Dinesh H. Wellawa, Brenda Allan, Aaron P. White, Wolfgang Köster

**Affiliations:** 1 Vaccine & Infectious Disease Organization-International Vaccine Centre, University of Saskatchewan, 120 Veterinary Rd., Saskatoon, SK S7N 5E3, Canada; dinesh.wellawa@usask.ca (D.H.W.); brenda.allan@usask.ca (B.A.); aaron.white@usask.ca (A.P.W.); 2 Department of Veterinary Microbiology, Western College of Veterinary Medicine, University of Saskatchewan, Saskatoon, SK S7N 5B4, Canada

**Keywords:** *Salmonella*, iron homeostasis and regulation, chicken, pathogenicity, iron transport

## Abstract

Iron is an essential micronutrient for most bacteria. *Salmonella enterica* strains, representing human and animal pathogens, have adopted several mechanisms to sequester iron from the environment depending on availability and source. Chickens act as a major reservoir for *Salmonella enterica* strains which can lead to outbreaks of human salmonellosis. In this review article we summarize the current understanding of the contribution of iron-uptake systems to the virulence of non-typhoidal *S. enterica* strains in colonizing chickens. We aim to address the gap in knowledge in this field, to help understand and define the interactions between *S. enterica* and these important hosts, in comparison to mammalian models.

## 1. Introduction

The genus *Salmonella* is composed of two species, *Salmonella enterica* and *Salmonella bongori*. *Salmonella enterica* is subdivided into six subspecies; *enterica* (I), *arizonae* (IIIa), *diarizonae* (IIIb), *houtenae* (IV), *salamae* (II) and *indica* (VI), based on antigenic properties (somatic (O), flagellar (H1, H2) and capsule (K) antigens) and biochemical properties [1,2,3]. *Salmonella bongori* predominantly resides as commensal in ectotherms and except for a few incidences, mammalian infections are rare [4,5]. It is hypothesized that adaptation to different niches paved the pathway for speciation of *S. enterica* and *S. bongori* from a common ancestor by means of gene gain, gene loss and conjugation events [6,7,8]. *Salmonella enterica* contains >2600 serovars which can infect insects, wild birds, reptiles and mammals. A significant proportion of human salmonellosis (>99%) are caused by serovars under subspecies I (*enterica*) hence it is the most important category in terms of public health. Clinical manifestation of salmonellosis can vary among serovars. The gastroenteritis-causing strains are collectively known as non-typhoidal *Salmonella* (NTS) strains and this review mainly focuses on NTS. Gastroenteritis is associated with intestinal inflammation and diarrhea without fever in general. NTS strains have the capacity to infect broad livestock species, yet chickens (*Gallus gallus domesticus*) are known to be a major reservoir. This is supported by epidemiological data indicating that poultry represent a major epicenter for human salmonellosis (non-typhoidal) globally (Table 1) [9,10].

### 1.1. Iron Homeostasis by Salmonella in a Nutshell: Regulation and Iron-Uptake Systems

**Figure 1:**
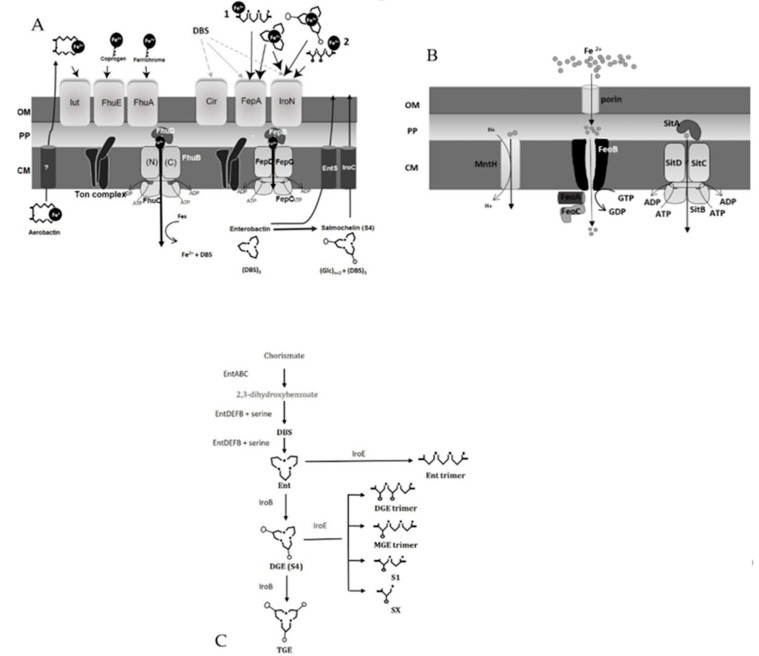
Schematic representation of iron-uptake systems in non-typhoidal Salmonella strains. (**A**) Fe^3+^ uptake systems. Enterobactin, salmochelin and aerobactin are secreted (e.g., through EntS and IroC) to sequester Fe^3+^ and then bind to their cognate receptors in the outer membrane (OM). Coprogen and ferrichrome are other ferric iron chelators present in the environment. Energy is generated through the proton motive force (PMF) in the cytoplasmic membrane (CM) and transduced to the receptor by the Ton complex (TonB-ExbB-ExbD). The energized receptor undergoes a conformational change which opens the pathway to mediate uptake of the iron-loaded siderophores into the periplasm (PP). The iron-liganded siderophores bind to periplasmic binding proteins (FhuD, FepB) which then shuttle them through ABC family permeases into the cytosol. 1,2 represent linearized forms of enterobactin and salmochelin respectively. (**B**) Fe^2+^ uptake systems. Ferrous iron in aqueous medium travels though porin channels in the OM according to the concentration gradient. FeoABC is specific for Fe^2+^ uptake. Both MntH and SitABCD are divalent metal transporters. (**C**) Forms of siderophores. Cyclic forms of enterobactin and salmochelin are hydrolyzed by the *iro* gene cluster to produce linearized forms of iron chelators.

Iron is an indispensable element for *S. enterica*. Key enzymes involved in bacterial metabolism depend on iron as a cofactor including DNA synthesis and repair enzymes [16]. Due to its transitional nature, iron can be either Fe^2+^/Fe^3+^ at physiological pH (7.2). In anaerobic environments, Fe^2+^ can be dominant over ferric iron, while Fe^3+^ can be abundant in aerobic conditions. *Salmonella* has established various mechanisms to internalize iron depending on its availability. In this review, we will discuss several important iron-uptake systems available in chicken-associated NTS strains (Figure 1). For more detail about iron homeostasis in bacteria in general, readers are directed to several references [16,17,18,19,20,21].

### 1.2. Ferric Uptake Regulator (Fur)-Mediated Regulation of Iron Uptake, Storage and Utilization

In addition to its innumerable beneficial effects, iron also catalyzes toxic metabolites such as superoxides, hydroxyl free radicals through Haber-Weiss and Fenton reactions in vivo which can damage bacterial DNA, iron-sulfur clusters, hence being harmful unless regulated [22,23]. The regulation is mainly under an auto-regulated protein called ferric uptake regulator (Fur) [24]. Fur acts as a repressor for most promoters related to iron uptake [25,26,27]. Under iron rich conditions, Fur binds to Fe^2+^, which causes Fur dimerization and subsequent binding to a consensus DNA sequence called the “fur box” (GATAATGATAATCATTATC), often present in promoter-containing regions. Binding overlaps the RNA polymerase (RNAP) binding sequence in the promoter region of iron-regulated genes [28]. This, in turn, hinders transcription of genes by the RNAP. Under iron-depleted conditions, Fe^2+^ dissociates from the dimer, the blockade for RNAP is removed, and iron-regulated genes are expressed. Apart from serving as a direct transcriptional repressor, Fur positively regulates iron storage and iron utilization genes via small RNAs called RyhB (*E. coli*) or its homologues (RfrA/B in *Salmonella*) [29,30]. For an example, under iron-rich conditions Fur upregulates iron storage proteins called bacterioferritins in *E. coli* via RyhB [31]. First, Fur-Fe^2+^ represses *ryhB* transcription and downregulates RyhB accumulation in the cell. Low intracellular RyhB concentration in turn alleviates RyhB-mediated destruction of mRNA transcripts and leads to the upregulation of iron storage proteins. The network of interacting partners by RyhB and its homologs have added more complexity to the Fur mediated iron regulation and interactosome of these RNAs are under active research [32].

### 1.3. Uptake of Ferric (Fe^3+^) Iron via Siderophores

Fe^3+^ is insoluble and often sequestered by host proteins (i.e., hemoglobin, transferrin, lactoferrin) or bound in complexes (Fe(OH)_3_) outside the host. *Salmonella* secrets high-affinity iron-binding molecules called siderophores (500–1000 da) to hijack Fe^3+^. Two siderophores belonging to the catecholate type are well-characterized: enterobactin and salmochelin. Enterobactin is nature’s superglue for Fe^3+^ which forms an incredibly stable complex with ferric ion at K_f_ = 10^49^ (K_f_ = formation constant) [33]. Chemically it is designated as the cyclic trilactone of *N*-2,3-dihyroxybenzoyl-l-serine. *N*-2,3-dihyroxybenzoyl l-serine (DBS) is the building block of enterobactin which undergoes cyclization to accommodate iron by six coordinated oxygen atoms in three DBS units. DBS itself can scavenge Fe^3+^ with low affinity [34]. Salmonella uses nonribosomal peptide synthesis pathways (NRPS) encoded by *entBCDE* (Figure 1C) to generate enterobactin in the cytoplasm which is then exported by EntS located in the inner membrane [35]. Except for some chicken-specific *Salmonella* serovars, all other chicken-associated-*Salmonella* produce enterobactin [36]. Enterobactin can be further linearized due to the action of hydrolase enzymes (IroE) located in the bacterial periplasm before secretion (Figure 1C). The linearized forms of the enterobactin (Ent-trimer, Ent-dimer) retain the ability to scavenge ferric iron, but with reduced affinity compared to its cyclic form (K_f_ = 10^43^) [33]. Once the secreted enterobactin and linearized forms are iron-loaded, they are taken up by their cognate receptors in the Salmonella outer membrane. Cyclic and linearized forms of enterobactin (ex; Ent-trimer) specifically bind to FepA. Evidence has suggested that enterobactin break down products like DBS, can be transported via Cir, FepA and IroN once loaded with Fe^3+^ [37]. These receptors share sequence similarity and follow the same general structure [18]. They are composed of a 22 antiparallel stranded β-barrel (which forms the channel) and an *N*-terminal globular domain referred to as the “plug” or “cork”. The energy generated by the proton motive force in the inner membrane is coupled to the outer membrane receptors via the TonB-ExbB-ExbD complex to achieve siderophore internalization (passage through the upper binding pocket), then migration through the channel (plug undergoes conformational changes) into the periplasm. Internalized iron is then released by degradation of enterobactin using Fes enzymes located in the cytoplasm.

Enterobactin can be glycosylated by a glycosyl transferase enzyme, IroB, to form salmochelin [38]. Glycosylation affixes glucose molecules to enterobactin thus forming the more hydrophilic salmochelin. It has been hypothesized that salmochelin is produced to counteract iron starvation mounted by the host. This has been supported by the observation that salmochelin is a better iron scavenger than enterobactin in presence of serum albumin and also it is not bound by the mammalian innate molecule lipocalin 2 (Lcn-2) which captures apo-enterobactin or Fe^3+^-enterobactin to impede iron scavenging by bacteria [39,40]. Lcn-2 is secreted by phagocytic cells (macrophages, neutrophils) and epithelial cells during host’s inflammatory response. Glycosylation of the enterobactin moiety sterically hinders the binding capacity of Lcn-2 and therefore salmochelin is considered a “stealth” siderophore. IroB can sequentially synthesize several versions of salmochelin termed mono glycosylated enterobactin (MGE), di-glycosylated enterobactin (DGE/S4) and tri-glycosylated enterobactin (TGE) [38,39]. Work done by Lin et al., 2005 has further demonstrated that the periplasmic enzyme IroE can linearize salmochelin to linear trimer (linearized TGE/S3-not shown in the Figure 1C), linear dimer (DGE/S2), MGE trimer, linear *C*-glycosylated (DBS)_2_ (S1) and linear monomer (SX) in vitro [39]. Also, the authors showed that IroD, a cytoplasmic esterase, can degrade the salmochelin forms into its building blocks (DBS) thus releasing the iron into the bacterial cytoplasm [40]. Salmochelins have high specificity for outer membrane receptor IroN and are subjected to TonB-dependant uptake like other siderophores.

Some NTS serovars produce aerobactin, a mixed type of siderophore known as citrate-hydroxamate type. Aerobactin is synthesized by a NRPS pathway utilizing enzymes encoded in the *iucABCD* operon. During synthesis, l-lysine is first converted to *N*^6^-acetyl-*N*^6^-hydroxy-l-lysine and then complexed into a citric acid backbone [41]. The iron complex formation constant of aerobactin (K_f_ = 10^23^) is weaker than that of enterobactin [42]. Aerobactin follows the same rule as catecholate-type siderophores regarding its uptake (Iut receptor) and TonB-dependant transport into the bacterial periplasm. Once in the periplasm, aerobactin is transported through the binding-protein-dependent ABC transport system FhuBCD [43]. FhuBCD also mediates the energy-dependant uptake of ferrichromes and coprogen from the environment (Figure 1A) [43].

A less common class of siderophores which can be found in *Salmonella* serovars are phenolate type siderophores such as yersiniabactin (Ybt). Ybt is abundantly produced in *Yersinia* species encoded by a genomic island called high pathogenicity island 1(HPI) [44]. HPI 1 is absent from most *Salmonella enterica* serovar subspecies 1 [44] and hence its distribution in *Salmonella* serovars is low. Seven proteins (HMWP1, HMWP2, YbtD, YbtE, YbtS, YbtT and YbtU) have been described in Ybt synthesis from the precursor isochorismic acid in *Yersinia* species. The final product is a four-ring structure composed of salicylate, one thiazolidine and two thiazoline rings. Ybt shows a higher affinity for Fe^3+^ (K_f_ = 10^36^) than aerobactin, hence it is a potent iron chelator. Once loaded with iron, yersiniabactin is taken up by the Psn/FyuA receptor in the outer membrane and then shuttled through the YbtPQ ABC transporter across the inner membrane (not shown in the figure).

### 1.4. Uptake of Ferrous Iron (Fe^2+^) via FeoABC, SitABCD and MntH

Ferrous iron is water-soluble and can readily pass through the outer membrane porin proteins into the periplasm following the concentration gradient. Once in the periplasmic space, Salmonella can take up Fe^2+^ via 3 systems: FeoABC, SitABCD and MntH. FeoABC belongs to a family of transporters that have high specificity for Fe^2+^. For the FeoABC system, the FeoB permease forms a channel in the inner membrane and FeoA and FeoC interact with FeoB in the cytoplasm. The *N*-terminal, cytoplasmic portion of FeoB contains a G-protein domain which can perform GTP binding and hydrolysis. Therefore, Feo-mediated Fe^2+^ uptake is coupled to GTP hydrolysis and signal transduction. For the latest structure and biology of the FeoABC system, readers are directed to two recent articles [45,46].

SitABCD is an ABC transporter family protein complex allowing the passage of primarily Mn^2+^ in alkaline pH but capable of transporting Fe^2+^ with low affinity [47]. Kehres et al., 2002 showed that SitABCD of Salmonella Typhimurium only transported Fe^2+^ when the concentration of Fe^2+^ reached 1 µM or higher in vitro [47]. MntH was also dominant in transporting Mn^2+^ rather than Fe^2+^. It was evident that uptake of Mn^2+^ was independent of pH, while Fe^2+^ transport increased by the acidic pH [47]. Further, it was revealed that affinity for Fe^2+^ to MntH was much lower than to SitABCD and only transported ferrous iron when it reached a concentration of higher than 1 µM in vitro [47]. Since the free, labile iron level is believed to be extremely low in biological fluids (<10^−18^M) and tissues, the role of SitABCD and MntH in ferrous iron transport is hypothesized to be of relatively minor significance compared to Feo-mediated iron uptake. The FeoABC system is recognized as the main ferrous iron transporter for many Enterobacteriaceae [48].

## 2. Emergence of Chicken-Associated Invasive NTS: The Iron Link

NTS strains are mainly asymptomatic colonizers in adult chickens, but strains of certain serovars can be fatal when infecting day-old chicks [49,50]. The major chicken-associated NTS serovars with potential to cause human epidemics are listed in Table 2. In countries belonging to the European Union (EU), the majority of breeders and layers were infected with *Salmonella* Enteritidis (SEn) while broilers were dominantly colonized by *Salmonella* Virchow (SVr) [51]. In contrast to the EU countries, *Salmonella* Kentucky (SKn) has been the predominant serovar isolated from poultry products in North America [10,52]. Generally, there is a high genetic synteny among NTS serovars (listed in Table 2) of chicken origin at core genomic levels [53]. Table 2 has only listed some the genetic differences which may be linked to virulence in chickens or humans.

**Figure 2:**
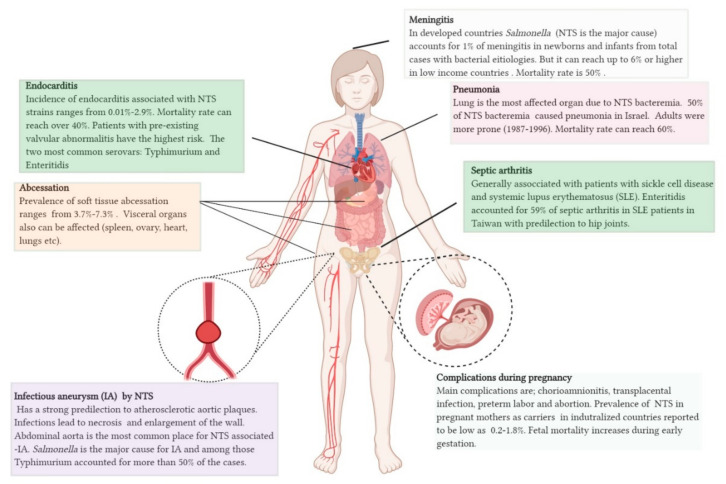
Bacteremia-induced complications by non-typhoidal *Salmonella*. Generally, 5% of gastroenteritis cases develop into bacteremia-associated complications in immunocompetent people. However the burden of NTS bacteremia is higher in immunocompromised patients and children under 5 years old (can reach up to 34%). Data related to epidemiology has been obtained from a variety of published case reports and outbreak analysis. [99,100,101,102,103,104,105,106,107,108,109,110].

The chicken host has been a hotspot for shaping new NTS pathotype strains that can cause extraintestinal diseases in humans due to bacteremia, often with antimicrobial resistant (AMR) phenotypes. NTS bacteremia can lead to severe inflammation within different organs, leading to organ dysfunction and sometimes death (Figure 2). In these more systemic infections, antibiotics are required for successful treatment. *Salmonella* Heidelberg (SHb) and SVr are among the top-four NTS serovars with highest invasiveness indices (proportion of bacteremia from total isolates) globally for which chickens act as a reservoir [75,76,77,78]. Apart from that, *Salmonella* Typhimurium (STm) and SEn are cumulatively responsible for the highest human epidemics globally with potential to cause blood-borne infections [79]. Comparative genomic analysis has predicted that *Salmonella* pathogenicity islands (SPI), adhesin molecules (fimbriae, invasins), secretion systems, virulence plasmid (spv), toxins, multidrug resistant genomic islands and colonization factors have a role in causing blood-borne infection in humans [52,53,57,58,80,81]. Another important virulence trait that has been overlooked in NTS serovars is iron uptake. As summarized in Table 2, there is a general trend in strains of important NTS serovars to acquire additional iron-uptake systems. Kajanchi et al. (2017) reported that a significant number of STm strains, isolated from chickens, turkeys and humans, carried ColV plasmids which encoded genes for divalent metal uptake (*sitABCD*) and Fe^3+^ uptake via synthesis, secretion and translocation of aerobactin (*iucABCD*, *iut*) [62]. The plasmid encoded *sitABCD* was phylogenetically distinct from the chromosomally encoded loci. The effect of having two *sitABCD* operons for clonal expansion and/or virulence is still unknown. ColV plasmids have been associated with SKn strains and to a lesser extent with SHb strains in the USA that were isolated from poultry [55]. For SKn, there was a significant fitness defect in colonizing the chicken cecum in strains lacking pColV [55]. In addition, systemic dissemination and the ability to cause splenic lesions was reduced in pColV null background compared to the pColV positive strain, indicating that genetic factors carried in pColV plasmids are important virulence determinants during extraintestinal disease [55]. SKn is an emerging pathogen which can cause blood-born infections in humans [82,83,84], thus ColV plasmid-encoded factors including iron uptake functions most likely contribute to overall virulence.

The aerobactin operon (*iucABCD*), also carried on pColV plasmids, is of particular interest, because normally its prevalence is low in most Salmonella [42]. Aerobactin-producing NTS serovars (SEn, STm, SVr,SIn etc) were highly associated with human salmonellosis caused by ingestion of contaminated poultry products in Spain [85]. In some reports, it has been documented that aerobactin production is exclusively linked to blood-born infection rather gastroenteritis, as aerobactin-producing NTS serovars were exclusively isolated from human blood [86,87]. In fact, some of the properties of aerobactin can provide NTS serovars a better survivability during systemic dissemination, even though affinity to Fe^3+^ of aerobactin is lower than most other siderophores. Some of these features include: higher transfer rate of Fe^3+^ from transferrin receptors to aerobactin in the serum, higher solubility, low wastage of resources during aerobactin production (recycled) and rapid secretion out of the cells to be available for ferric uptake compared to enterobactin, which tends to accumulate in the inner-membrane [42]. The iron in the mucosal surface of the gastrointestinal tract is mainly bound by lactoferrins which has a high affinity for iron (K_f_ = 10^20^) like transferrin [88,89]. Therefore, secretion of additional siderophores such as aerobactin may provide NTS strains a competitive advantage for multiplication and invasion into the gastrointestinal tract. In addition, aerobactin is not bound by Lcn-2 which will provide a defense against Lcn-2-mediated iron starvation during inflammation. So, in the bottom line, aerobactin can be involved not only in the systemic phase of infection but also in enteric infection. The pColV plasmids are well-distributed among *E. coli* strains and it is believed that chicken-associated NTS strains may have acquired the pColV from an avian pathogenic *E. coli* (APEC) strain. APEC strains cause high morbidity and mortality in chickens (colibacillosis) due to their ability to cause septicemia [90]. Dozois et al., 2003 showed that among pathogen-specific gene clusters expressed in APEC strains, both aerobactin and salmochelin were important for virulence in chickens [91]. Further, significant reduction of colibacillosis-associated pathology was observed in an aerobactin-knockout APEC strain carrying ColV plasmids [92]. In a similar manner the hypervirulent *Klebsiella pneumoniae* strain solely uses aerobactin to confer its hypervirulent phenotype which leads to septicemia in humans [93]. By all these means, acquiring aerobactin production may indeed cause the chicken-associated NTS serovars to become more virulent once infected in humans. There are number of other genetic factors encoded on pColV plasmids which can contribute to virulence, including the *iss* gene associated with increased serum survival in APEC strains [94]. Therefore, experimental approaches will be necessary to study the role of aerobactin encoded on pColV regarding virulence of NTS serovars in chickens and humans.

A recently emerging poultry-associated multidrug resistant *Salmonella* Infantis (SIn) lineage, harbored yersiniabactin secretion systems *(irp*) on pESI like plasmids [71,95]. As mentioned earlier, yersiniabactin is rarely present in Salmonella strains and its role is unknown regarding the existence in chicken-associated NTS strains. Yersiniabactin can sequester copper iron apart from ferric, to form a stable complex (yersiniabactin-cupric) which resists proteasomal degradation. In a series of experiments conducted by Chaturvedi and colleagues, they were able to show that the yersiniabactin-cupric complex neutralized superoxide (super oxide dismutase-like activity) generated in phagosomes which gave uropathogenic *E coli* bacteria, a survival advantage in vitro and in vivo [96,97]. This new paradigm for the role of yersiniabactin in virulence is highly applicable to NTS serovars, because *Salmonella enterica* species do need to resist copper (Cu^2+^) accumulation inside macrophages for the survival [98]. Once accumulated in the cytoplasm of macrophages, Cu^2+^ oxidized into a Cu^1+^ which is toxic to bacteria. So, co-expression of yersiniabactin and catecholate siderophores (enterobactin, salmochelin) in the SIn strain may provide a survival advantage by facilitating iron acquisition as well resistance to copper-mediated toxicity.

The acquisition of siderophore secretion and metal iron-uptake systems in chicken-associated NTS serovars might be linked to their invasive phenotypes in humans but more studies are needed to confirm their role. Whether they are important for the pathogenesis in chickens remains a question to be answered.

## 3. Iron Uptake in NTS Virulence: Chicken vs. Mammalian Models

**Figure 3:**
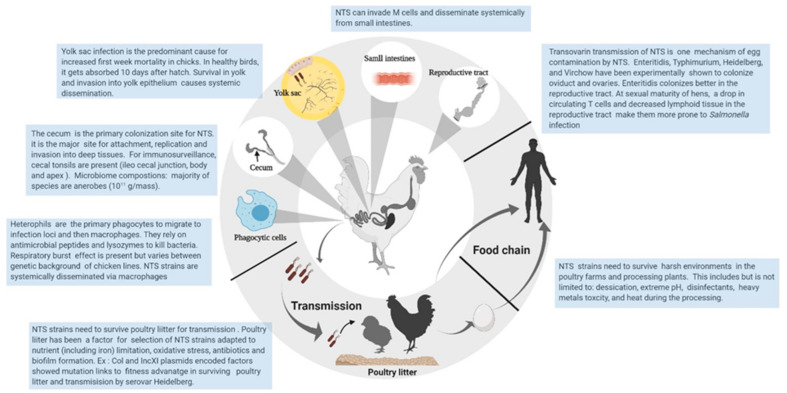
Interaction of non-typhoidal *Salmonella* strains with chicken and the environment. The role of iron-uptake systems during key steps of *Salmonella* life cycle illustrated here needs to be investigated in chicken models in future [50,114,115,116,117,118,119,120,121,122].

Most of our understanding related to the role of iron-regulated gene clusters in *Salmonella* pathogenesis has derived from experimental infection with *Salmonella* Typhimurium (STm) using mouse models and mammalian cell culture assays. Due to differences in how pathogens interact with avian environments, we cannot directly extrapolate this information to chickens [111,112]. The relationship of virulence with various iron-uptake systems in pathogenic bacteria including *Salmonella enterica* species has been extensively reviewed [16,17,18,19,20,21,113]. Unfortunately, limited data in chicken models and avian cell lines remains a barrier to understanding the host–pathogen interactions of the iron-uptake system in Salmonella serovars. Here we discuss the potential role of iron-uptake systems in NTS serovars towards infection and colonization in chickens compared to mammals. Some of the gaps in knowledge which need to be addressed in poultry are summarized in Figure 3.

### 3.1. Feo-Mediated Fe^2+^ Uptake Involved in Rapid Colonization of the Gut and Systemic Spread

To identify differentially expressed gene profiles of STm isolated during colonization of the lumen of the chicken cecum (compared to in vitro cultures), Harvey et al., 2011, detected the upregulation of the *sitABCD* operon [123]. In contrast, the major Fe^2+^ uptake facilitator, the FeoABC system, was not differentially expressed during the same experiment. However, these researchers only assessed gene expression at 16 hours post-infection in newly hatched chicks so they may not have been able to capture the full spectrum of iron-regulated gene expression over time [123]. The *sitABCD* gene cluster was a major virulence factor in an avian pathogenic *E. coli* (APEC) strain causing colibacillosis in a chicken air sac model [124]. Evidence suggested that manganese uptake was more important than the Fe^2+^ uptake during extraintestinal phase in APEC strains [124]. In contrast, both Mn^2+^ and Fe^2+^ uptake contributed to the full virulence of STm to cause typhoid disease in mice [125]. Portillo et al., 1992 estimated that 1 µM of free Fe^2+^ prevailed inside the STm containing vacuole within Madin-Darby canine kidney cells and it was sufficient for replication, for at least 8 h of infection [126]. This suggested that Fe^2+^ iron uptake might be more important than Fe^3+^ in the initial stages of STm establishment in the gastrointestinal tract. Supporting this hypothesis, Tsolis et al., 1996, showed that the lack of the Feo system significantly reduced the fecal shedding of STm in mice (C57BL/6) at day 4 post-challenge while a Fe^3+^ uptake null strain was recovered at a level similar to the wildtype [127]. In line with these findings, Costa et al., 2017 showed that Feo-mediated iron uptake provided a fitness advantage for STm, during gastrointestinal colonization (fecal shedding) via intragastrical route in a streptomycin-pretreated mouse (C57BL/6) colitis model at 2 days post-infection [128].

Similar to mammals, chickens mediate Fe^2+^ egress from macrophages by expressing NRAMP-1 (Natural Resistance-Associated Protein 1) in the phagosomal membrane [129]. The action of NRAMP-1 is thought to limit the free, labile iron pool available to intracellular pathogens [130]. Thus, it is very likely that Feo-mediated ferrous iron uptake plays a crucial role for Salmonella to establish systemic infections in chicken. NRAMP-1 expression has been linked to Salmonella-resistance in certain chicken genetic lines (White Leghorn W1) [131]. The susceptible chicken line (CC) had a conservative mutation in the amino acid residue located at 223 (Arg^223^→Gln^223^) of NRAMP-1, which was highly predictive of a functional anomaly in the NRAMP- 1 protein [131]. Consistent with this finding, authors observed that only 15% birds survived to a parenteral challenge of STm in the susceptible chicken line (CC) 7 days post-infection while almost all birds survived in the resistant chicken line [131]. However, the mortality rate of the susceptible chicken line was comparable to the resistant chicken line beyond day 7 post-infection irrespective of the NRAMP-1 status [131]. This reflected that Fe^2+^ starvation in presence of a functional NRAMP-1 certainly did limit rapid systemic spread of the STm in chicken but bacteria somehow adopted the new iron status in chicken and survived, pertaining their virulence during persistent infection. Future studies are needed to examine the iron distribution during *Salmonella* pathogenesis in chicken (cecal colonization and extraintestinal dissemination) and how this will shape overall regulation of iron-uptake systems in NTS serovars.

While further experiments are warranted to investigate the role of Fe^2+^uptake in relationship to the NRAMP-1 status in chicken lines, mouse models of infections have provided some insight into the interplay between NRAMP-1 and Feo-mediated iron uptake. Feo-mediated iron uptake provided a competitive advantage during persistent infection of STm (SL1344-calf virulent isolate) in both NRAMP-positive and -negative backgrounds of mice [132]. Authors observed that a Δ*feo* STm strain was significantly reduced in its ability to colonize deeper tissue in the gut such as Peyer’s patches (PP), mesenteric lymph nodes (MLN), as well as liver and spleen during a mixed infection [132]. Mice were orally challenged resembling natural infection with *Salmonella*. In the same study, it was documented that the lack of Feo-mediated Fe^2+^ uptake affected the overall iron homeostasis in a STm strain during a single infection challenge model [132]. The study revealed that the Δ*feo* STm strain compensated the requirement of iron by upregulating siderophore-mediated Fe^3+^ uptake (enterobactin, salmochelin) systems during systemic infection (liver and spleen) [132]. Interestingly, this upregulation of siderophore-mediated ferric uptake resulted in increased bacterial burden in the liver and spleen during persistent infection in NRAMP^+/+^ mice [132]. There are growing numbers of evidence indicating that Salmonella preferentially resided in hemo-phagocytosed macrophages in the liver and spleen during infection [133,134,135]. One plausible explanation for this might be the abundant source of iron that Salmonella can exploit during degradation of erythrocytes (Fe^3+^/Fe^2+^) in those macrophages. Hence hypersecretion of siderophores may benefit growing *Salmonella* under such conditions. Expression of iron-uptake systems certainly may differ among different types of tissues the bacterium has to encounter or might vary due to host responses. For example, transferrin-bound iron (Fe^3+^) in the intestines provides a good source of iron for Salmonella and the uptake can be facilitated by the stress-induced norepinephrine hormone which is produced abundantly in the mesenteric organs both in chickens and mice [136].

**Highlights-1:**

(i) Feo-mediated ferrous iron uptake is important for rapid colonization by and systemic spread of *Salmonella* Typhimurium in NRAMP^+/+^ mice. We predict the same in chicken–NTS interaction.
(ii) Feo may not be essential for persistent infection in mouse models due to redundancy of various iron-uptake systems. This includes Mn^2+^ uptake via SitABCD and MntH, and uptake of siderophores.
(iii) NTS predilects to iron-rich hemophagocytes during systemic infection.

### 3.2. Siderophore Synthesis Is Important During Persistent Infection and Bacteremia

Iron restriction is well-studied related to antimicrobial properties of egg white in vitro [137,138,139,140,141,142]. Kang et al., 2006, showed that a Δ*entF* strain of SEn which was unable to produce a catecholate siderophore, was significantly attenuated in its ability to survive in egg albumen in vitro which suggested that siderophore production is an important virulence determinant during internal contamination of the eggs [143]. The egg is enriched with a variety of iron chelators such as ovotransferrin (in egg white) and phosphovitin (in yolk) hence it is very likely that potent ferric hijacking systems will benefit *Salmonella* in colonizing the eggs during transovarian transmission. Van Immerseel et al., 2010, proposed the hypothesis that stress-induced survival mechanisms governed by SEn led to egg-associated human outbreaks due to the fact that eggs possessed an arsenal of antimicrobial properties [144]. However, in-vivo gene expression studies did not identify iron-uptake systems as differentially expressed gene clusters during oviduct colonization or egg contamination [145,146]. Gene expression studies have been conducted using an intravenous challenge model which is an unnatural route of infection in hens. So, it might be possible that gene expression of *Salmonella* during intravenous challenge might be different compared to oral infection in hens. Siderophore-mediated ferric iron uptake has not been identified as a major virulence determinant during colonization in the gut and systemic infection in chicken, so far. There are not enough studies performed using iron-homeostasis-related mutants of Salmonella to investigate their role in infection, colonization and transmission in a chicken model. In a series of experiments executed by Rabsch et al., 2003, it was proposed that siderophore degradation product such as *N*-2,3-dihyroxybenzoyl-l-serine (DBS) will be more important in colonization and systemic spread in the absence of an active siderophore uptake system in chicken [37]. The authors confirmed this hypothesis in a mouse model of infection (intragastric route) using a *ΔfepA ΔiroN Δcir* strain of STm (SL1344) which was significantly attenuated in colonization of the cecum and systemic spread, which in contrast was not observed in a *ΔfepA ΔiroN* mutant (enterobactin and salmochelin uptake deficient). In a chicken model, SEn strains carrying *fepA iroN* mutation profiles behaved similarly as in mice indicating that siderophore uptake was not essential during early colonization events [37]. Interestingly, the authors concluded that in BALB/c mice who are intrinsically susceptible to *Salmonella* infection, salmochelin was not important to cause infection. All these data have to be used cautiously due to following reasons; (i) *N*-2,3-dihyroxybenzoyl-l-serine (DBS) is not occurring naturally in the environment. It needs to be synthesized (*entABCDE*) or liberated as a byproduct due to action of Fes and IroE (Figure 1) on enterobactin/salmochelin. So, if DBSs are important so is the siderophore synthesis. When uptake routes are blocked spontaneous breakdown of siderophores can be a rapid process. (ii) At a given time, siderophores and its degraded products (enterobactin, salmochelins, Ent-trimer, Ent-dimer, DGE-trimer, DBS etc.) can be present and this cocktail may have a biological role in vivo. For example, degradation to more soluble form such as DBS, enables *Salmonella* to internalize iron rapidly. The mixture of derivatives might also exhaust the immune system in mounting an effective antibody response (antibodies against one particular siderophore derivative will spare others in the mixture) [147,148]. (iii) The genetic background of the host organism will have a major effect on the outcome of animal experiments. For example, the importance of iron-uptake systems described in mice that are genetically susceptible or resistant to Salmonella has been contrasting [149,150]. This will most likely be applicable to chickens as well (Salmonella*-*resistant and -susceptible chicken lines). Another crucial factor is the age of the birds: e.g., chicks (weak immune system) vs. adult chickens.

Fe^3+^ uptake via FepB (periplasmic binding protein for some catecholate type siderophores) has been identified as an absolute requirement for the persistent infection in mice (Sv129S6-Nramp1^+/+^) with STm (SL1344) [151]. FepB is needed to shuttle Fe^3+^ bound to enterobactin, salmochelin or DBS (2,3-dihydrobenzoic acids), from the periplasm to the inner membrane transport components (Figure 1A). The *ΔfepB* of STm dramatically lowered the bacterial recovery below the detection limit in most of the tissues examined in mice (cecum, MLN, PP, liver and spleen) [151]. Most importantly, the authors in these studies showed that siderophore synthesis (enterobactin, salmochelin) played a significant role in gastrointestinal colonization and systemic spread during persistent infection [151].

Salmochelin synthesis and export have been identified as major virulence factors during bacteremia in mice (C3H, Nramp^+^) measured by mortality after intraperitoneal injection of STm [152]. Parenteral injection of STm carrying a mutation in *tonB* which completely blocked all siderophore uptake has previously been shown to significantly increase the LD50 in mice compared to the challenge with wildtype STm [127]. Further, in a study which analyzed differentially expressed genes in STm-SL1344 by transcriptomic and proteomics techniques, enterobactin synthesis and uptake genes were highly upregulated during systemic infection in a mouse (C57BL/6) model [153]. Most interesting finding of that study was, in addition to enterobactin, salmochelin-related genes were upregulated in immune-deficient mice background (deficient in ROS generation) but not in wildtype mice background [153]. In the same study high bacterial growth has been observed in spleen of immune-deficient mice which may have been linked to a high demand of iron for growth of STm [153]. It is well-documented that salmochelin provides a defense against Lcn-2-mediated enterobactin chelation by the host during inflammation (mouse colitis model) [154]. Hence it is possible that mice with deficiency in respiratory burst effect, may rely on antimicrobial mechanisms such as more Lcn-2 secretion to limit *Salmonella* replication in phagocytic cells. Also, serum is considered as an extremely low iron compartment for pathogens in vertebrates [155]. Serum iron is mostly bound to transferrin, albumin and ferritins. In the presence of serum albumin, enterobactin is not considered as an efficient iron chelator as it is rapidly cleared [156,157]. Hence secretion of stealth siderophores (aerobactin, salmochelin and yersiniabactin) will be beneficial for NTS serovars during bacteremia.

The extracellular fatty acid-binding protein (ExFABP) of chickens has been identified as the chicken equivalent of Lcn-2 [158]. Its overall structure is similar to Lcn-2 yet it has a more extended positively charged calyx (which is the binding pocket for ligands) with two binding specificities: one for siderophores and the other for lysophosphatidic acid [158]. Interestingly, the calyx of Ex-FABP accommodates one form of salmochelin, mono- glycosylated enterobactin (MGE/S1) which is not normally bound by Lcn-2 [158]. Lcn-2 cannot bind to any salmochelin derivatives. So, the “chicken lipochalin-2” seems to be more potent in withholding iron compared to Lcn-2 during *Salmonella* infection. There is ample evidence for expression of Ex-FABP in the cecum associated with inflammation of day-old chicks when infected by NTS [159,160]. Chicken egg white which has antibacterial properties against *Salmonella* in vitro also contains Ex-FABP [145]. Adult chickens generate a more tolerogenic response towards non-typhoidal *Salmonella* (NTS) infection [161,162]. The inflammation induced in adult chicken is transient yet sufficient enough to contain the bacteria in the gut while some may spread systemically to colonize spleen and liver. Significant inflammation in the liver and spleen has not been observed in more mature birds except for follicular lesion [161]. The lack of marked inflammatory response in adult chicken towards NTS infection is an indication that some of the stealth siderophore secretion might not be essential during the colonization process compared to mammals.

**Highlights-2:**

(i) Ferric iron uptake mechanisms are important for persistent infection.We predict similar results for chicken as those found in mouse models because bioavailability of iron is expected to be low in most compartments of the host.
(ii) Aerobactin, salmochelin and yersiniabactin provide a serum resistance during bacteremia and systemic infection. This may explain the siderophore link towards chicken-associated virulent NTS serovars.
(iii) The role of stealth siderophores of NTS in adult chickens during colonization may be nonessential due to tolerogenic response.

## 4. Opening the Pandora’s Box of Gallus-Iron-*Salmonella* Interaction

Iron uptake is a primary virulence factor for *Salmonella*. But how each iron-uptake system partakes in pathogenesis in a chicken model still needs a thorough investigation. This is intriguing because chickens are the major reservoir for Salmonella; yet we know least about its interactions with the host. We want to highlight some of the important aspects which need to be addressed in future experiments using chickens as model related to the Gallus-Iron-Salmonella interaction. This will certainly lay a platform to discuss the potential for developing therapeutics targeted at iron homeostasis in *Salmonella*.

### 4.1. Nutritional Immunity Status in Chicken during Salmonella Infection

Nutritional immunity is defined as part of the host’s innate immune response to withhold essential nutrients, including iron, from invading pathogens [163]. The interplay between iron-withholding mechanisms in chicken and iron homeostasis in Salmonella during pathogenesis is largely unknown. The interaction between siderophores and extracellular fatty acid binding protein (ExFABP), which is part of chicken-iron-withholding strategy, has recently been well-documented in eggs [164]. A study revealed that SEn has to synthesize stealth siderophores such as salmochelin to overcome iron starvation induced by ExFABP (chelation of Fe^3+^-enterobactin) in egg white in vitro [164]. Ovotransferrin, synthesized by oviduct cells, is a transferrin family protein which transports iron into the growing embryo. It is the major constituter of egg albumin. The iron complex formation constant of ovotransferrin (iron affinity of the C lobe is 10^18^ and N lobe is 10^14^ ) is low compared to most siderophores secreted by Salmonella hence iron restriction is not the major mechanism behind its antibacterial effect [165]. Egg yolk is the major iron store for growing embryos and almost all iron is bound to phosvitin. The affinity of phosvitin to iron is comparable to ovotransferrin (K_f_ = 10^18^) [166]. So, Salmonella can rely on enterobactin (K_f_ = 10^49^) rather on the expression of stealth siderophore to hijack iron from phosvitins, unless ExFABP is expressed in sufficient amounts. However, currently there is no evidence that stealth siderophores are indeed expressed to counteract ExFABP-mediated nutritional immunity in chickens during colonization in various tissue in vivo.

Adaptation to an iron-deficiency status in humans plays an important role in resistance to bacterial and viral infections [167]. The response is also termed as hypoferremia of inflammation or anemia of inflammation (AI). The key player for hypoferremic response is recognized as hepcidin, the master regulator for iron metabolism in humans and it is believed to be hepcidin independent in chickens (chicken genome seems to lack hepcidin up to date) [168,169,170]. Inflammation caused by pathogenic invasion induces hepcidin secretion from liver [171]. Hepcidin mediates ferroportin (Fpn) degradation which inhibits iron efflux from macrophages and iron absorption from intestines [172]. Fpn degradation also affects hepatocytes which increases their ferritin levels and the ability to store accumulated iron. All these mechanisms lead to a significant drop in the serum iron level (hypoferremia). The low level of iron in the serum may limit bacteremia, yet current evidence suggested that the burden of NTS increased in systemic infection-related sites such as the spleen during hypoferremic response in mice [173,174,175]. Similarly, infection with chicken-specific serovars such as *Salmonella* Gallinarum and *Salmonella* Pullorum led to anemia of inflammation (AI) in chicken with increased bacterial burden in spleen and liver [176]. The increased *Salmonella* colonization in the “systemic sites” correlated with a spike in the iron content both in mice and chickens. The reason for such a spike of iron in spleen can be partly due to the accelerated red blood cell turnover rate triggered by inflammation induced hypoferremia response. It has been documented that in mammals the half-life of red blood cells decreased dramatically during AI response and led to increased destruction of red blood cells by macrophages in spleen and liver [177]. Since Salmonella can profit from the iron abundancy (Fe^2+^/Fe^3+^) in hemophagocytic cells [132], they may preferentially rely on a specific iron uptake system during AI. Supporting this hypothesis, the African lineage of iNTS (invasive NTS) strain *Salmonella* Typhimurium 313 (ST313) appears not to rely on salmochelin-mediated Fe^3+^ uptake during systemic infection in mice [178]. There is a strong association of African linage of iNTS strains with malaria parasites which increase intracellular iron levels in macrophages [179]. An abundance of the Fe ^2+^ pool may have inherently adapted the ST313 to reduce the expression of stealth siderophore uptake systems which are a metabolically demanding process to produce. There is currently no experimental data indicating the occurrence of AI in chicken during NTS infection. Broader host range serovars such as NTS strains colonize mainly the gastrointestinal tract without overt inflammation in adult birds. In such a situation, AI will not be profound. Virulence of NTSs varies according to serovars and chicken susceptibility depends on their genetic background and the age of the birds. For example, NTS such as STm and SEn do cause systemic inflammation during enteric infection in young chickens. They are also capable of infecting the yolk sac in young birds leading to the development of omphalitis. Yolk sac infections result in high mortality due to septicemia [180]. The role of iron-uptake systems of NTS, when the host undergoes hypoferremia needs to be investigated in a chicken model of infection. It will be especially important to examine iron distribution in compartments such as blood, liver, spleen and gastrointestinal tract of chickens following infection. Research on iron-regulated gene expression combined with proteomic studies is needed to assess how each iron-uptake system is regulated in parallel to anemia of inflammation.

### 4.2. Non-Canonical Function of Siderophores: Defense against Respiratory Burst and Immunomodulatory Function

There are growing number of evidences suggesting that siderophores have other biological functions apart from Fe^3+^ uptake [181]. One such alternative function is defense against oxidative stress provided by catecholate siderophores [182,183,184]. The mechanism behind the enterobactin-mediated defense against reactive oxygen species is currently been investigated. One of the mechanisms suggested that Ent-trimer (Figure 1C) which is the linearized molecule of enterobactin, participates in ROS scavenging by providing hydroxyl groups from the freed end of the backbone [185]. Generation of a robust respiratory burst is a key mechanism to kill Salmonella inside phagocytic cells [186]. In this regard, catecholate siderophore production will provide a survival advantage inside macrophages which is a major replication niche during systemic dissemination and colonization. Also, it may be plausible that synthesis of catecholate siderophores will be important irrespective of iron limitation inside phagocytic cells because of their diverse functions apart from iron scavenging. Some of the phagocytic cells in the chicken immune system do not induce a strong respiratory burst effect to *Salmonella*. The chicken lacks neutrophils yet has heterophils that are functionally equivalent to neutrophils. Heterophils are unable to synthesize myeloperoxidases and rely on a repertoire of antimicrobial peptides to kill bacteria instead of respiratory burst [114,187,188]. It has been documented that enterobactin inhibited the myeloperoxidases activity in *E coli* and provided a survival advantage in inflamed gut [184]. Hence it is important to investigate the interplay between enterobactin and heterophils during gastrointestinal colonization. Macrophages from Salmonella-resistant chicken lines (*SALI)* showed more pronounced respiratory burst effect while susceptible and inbred lines had low, variable level respectively [189]. So future experiments are warranted in chickens to investigate the role of siderophore-mediated defense against reactive oxygen and nitrogen species.

Holden et al., 2016, showed that siderophores produced by *Klebsiella pneumoniae* (enterobactin, salmochelin and yersiniabactin) can induce inflammation in lung epithelial tissue by stabilizing the hypoxia inducible factor-1α (HIF-1α) in C57BL/6 mice [190]. In a previous study, Holden et al., 2014, showed that enterobactin together with Lcn-2 can potentiate the induction of pro-inflammatory cytokines in cultured murine lung epithelial cells through chelation of iron [191]. These data are highly suggestive that siderophores can mount an inflammation in vivo. Inflammatory cytokines liberated will help to attract macrophages and dendritic cells to the infective loci and subsequent systemic spread. It will be interesting to investigate whether siderophores facilitate systemic infection by induction of inflammation at different colonization sites in chicken by NTS serovars. Enterobactin-mediated iron chelation has been documented to polarize the macrophage from M1 phenotype to M2 phenotype in bone-marrow-derived cells [192]. M2 phenotype of macrophages will safeguard intracellular pathogen such as Salmonella by avoiding generating an oxidative killing mechanism [193]. Chicken has low number of resident macrophages in organs and relies on bone-marrow-derived monocytes to migrate to the inflammatory loci for pathogen control [187]. Presence of distinct M1 (killing/towards inflammatory) and M2 (healing/towards adaptive response) phenotypes [194] of chicken macrophages is yet to be fully elucidated. Further studies are needed to unravel how *Salmonella* mediates iron homeostasis in infected chicken macrophages as this microenvironment may impose a different iron status during polarization [195].

## 5. Concluding Remarks

Our understanding of iron in infection and immunity remains close to its infancy due to the complex nature of the interaction and ever-growing *Salmonella* serovars found in nature. Concerning chickens as a reservoir, it will be pivotal to understand how iron-regulated genes of *Salmonella* are expressed during pathogenesis in a chicken model of infection (Figure 3). Enhanced detection of in vivo siderophore production during colonization in different chicken host niches in situ will be key in understanding their role in the future. Experiments are needed to address how iron metabolism and homeostasis in the chicken are regulated in response to NTS infection. There are other metal uptake systems (Mn2^+^, Cu, Zn2^+^) apart from iron uptake which are not well-characterized in a chicken model regarding their role in NTS colonization. We believe that these efforts to understand the involvement of iron homeostasis in pathogenesis of NTS will pave the way for the development of a successful therapeutic strategy in the poultry industry to limit chicken-associated *Salmonella* “spillovers” to humans and the environment.

## Figures and Tables

**Table 1:** Some of the global incidences of human salmonellosis linked to poultry.

Serotype	Source	Year(s)	Geographical Region	No of Cases ^a^	References
Enteritidis	Chicken (shell eggs)	2010	USA	1939 ^b^	CDC 2020 ^d^
Enteritidis Virchow	Chicken	2010 (from 2007)	Brazil	>260	[11]
Enteritidis Virchow	Chicken	2010	Taiwan	>1000	[12]
Stanley	Turkey (meat)	2011–2013	EU	710	[13]
Heidelberg	Chicken (meat)	2011	USA	190	CDC 2020 ^d^
Infantis Newport Lille	Chicks, ducklings (live)	2012	USA	195	CDC 2020 ^d^
Heidelberg	Chicken (meat)	2013	USA	634	CDC 2020 ^d^
Typhimurium	Chicks, ducklings (live)	2013	USA	356	CDC 2020 ^d^
Enteritidis	Chicken (eggs)	2014	EU	>400	[14]
Multiple NTS ^c^	Chicks, ducklings (live)	2014	USA	363	CDC 2020 ^d^
Multiple NTS	Chicks, ducklings (live)	2015	USA	252	CDC 2020 ^d^
Multiple NTS	Chicks, ducklings (live)	2016	USA	895	CDC 2020 ^d^
Typhimurium	Chicken (egg)	2015–2016	Australia	272	[15]
Enteritidis	Chicken (eggs)	2016–present	EU	1656	ECDC 2020 ^e^
Multiple NTS	Chicks, ducklings (live)	2017	USA	1120	CDC 2020 ^d^
Typhimurium	Chicken (salad)	2018	USA	265	CDC 2020 ^d^
Reading	Turkey	2018	USA	358	CDC 2020 ^d^
Enteritidis	Chicken (processed meat)	2017–2019	Canada	584	Public Health Service 2020 ^f^
Enteritidis	Chicks, ducklings (live)	2019	USA	1134	CDC 2020 ^d^

^a^ Number of reported incidences. ^b^ Estimated due to inadequate reporting. ^c^ More than 3 NTS serovars were involved. ^d^ According to the online data published by the Centers for Disease Control and Prevention in 2020 June (https://www.cdc.gov/Salmonella/outbreaks.html). ^e^ According to the online data published by the European Centre for Disease Control and Prevention in 2020, June (https://www.ecdc.europa.eu/en/infectious-diseases-and-public-health/salmonellosis/threats-and-outbreaks). ^f^ Public Health Services, Canada website (https://www.canada.ca/en/public-health/services/diseases/salmonellosis-salmonella.html). NTS: non-typhoidal *Salmonella*.

**Table 2:** Most prevalent chicken-associated NTS serovars with public-health risk.

*Salmonella* Serovar	Genetic/Phenotypic Signatures	Role Related to Virulence in Chicken or Human	References
Kentucky (SKn)	(1) Colicin production (pColV) (2) Salmonella genomic island 1 (SGI1) (3) RpoS regulated gene cluster: csg (curli), prpBCDE (propionate catabolism) (4) Lack of Saf and Sef fimbria (5) Additional iron uptake carried in pColV; siderophores- aerobactin & salmochelin, *sit* operon (Mn^2+^, Fe^2+^ uptake)	(1) Increased colonization in chicken gut (2) Multidrug resistant (MDR) including 3rd generation cephalosporin, ciprofloxacin resistant *, (3) Upregulated in chicken cecal explants (4) Decreased invasiveness in humans compared to other NTS (5) NDA (no data available)	[54,55,56]
Heidelberg (SHb)	(6) Type IV secretion (T4SS) (7) SopE (T3SS1 effector) duplication in the chromosome (8) Salmonella atypical fimbria (*safABCD*) (9) Additional iron uptake carried in pColV; siderophore-aerobactin	(6) Dissemination of antibiotics resistance and efficient survival in macrophages (7) Invasion into epithelial cells and induce inflammation (8) Only presented in the outbreak strain linked to human salmonellosis. (9) NDA	[55,57,58]
Typhimurium (STm)	(10) *Salmonella* genomic island 1 (SGI1) (11) *Salmonella* genomic island 4 (SGI4) (12) Plasmid encoded factors; *mig-5*, *rck*, *spv* (*Salmonella* plasmid virulence), *pef* , (13) Additional iron uptake in pColV; aerobactin, salmochelin *sit* operon (Mn^2+^, Fe^2+^)	(10) Sequence type DT104 showed Increased egg contamination compared to SEn phage type 4, contains ACSSuT ^#^ drug-resistant phenotype (11) Heavy metal resistant in DT104 (12) colonization in chicken gut, systemic spread (13) NDA	[59,60,61,62]
Typhimurium mono phasic variant (STmv) (DT193/DT120)	(14) Phase 2 flagellin not expressed (*fljBA* operon) (15) SGI-4 (16) Lack of *Salmonella* plasmid virulence locus, (17) Lack of Gifsy prophages	(14) Predicted to be an adaptation related to the expansion of reservoir host (15) resistant to heavy metals copper and zinc (16) less invasive in humans (17) NDA	[59,63,64]
Enteritidis (SEn)	(18) pSLA5 plasmid (19) Plasmid encoded factors; *mig-5*, *rck*, *spv* (*Salmonella* plasmid virulence), *pef* (20) Peg fimbria	(18) Associated with recent outbreaks in the EU. (19) Colonization in chicken gut, systemic spread (20) Generally unique to Enteritidis. Facilitates cecal colonization in chickens	[65,66,67,68]
Virchow (SVr)	(21) *Salmonella* Typhi colonization factor: TcfA (22) Novel SopE effector	(21) TcfA fimbriae provides tissue tropism in invasion into human cells. Role in chickens unknown (22) Associated with invasive nature of SVQ1 strain linked to outbreaks in Australia	[53,69]
Montevideo (SMv)	(23) Typhoid-associated virulence factors; TcfA fimbria, cytolethal toxin B etc.	(23) Predicted to increase tissue tropism and invasions in humans. Role in chickens unknown	[53,70]
Infantis (SIn)	(24) Plasmid-encoded factors;(pESI like); MDR; Extended spectrum beta lactamases (*bla*_CTX-M-65_) Additional ferric uptake system; yersiniabactin (*irp*), Fimbria: *E coli* K88, Infantis plasmid fimbriae (*ipf*)	(24) Associated in human outbreaks. Also, plasmid-encoded fimbria were contributed a colonization in the gastrointestinal tract of chicks	[71,72,73,74]

* Global pandemic strain of SKn with ciprofloxacin resistant, ST198-X1-SGI1 originated from chickens. ^#^ ACSSuT = ampicillin, chloramphenicol, streptomycin, sulfamethoxazole and tetracycline.
